# Effectiveness and Safety of Nivolumab in Child–Pugh B Patients with Hepatocellular Carcinoma: A Real-World Cohort Study

**DOI:** 10.3390/cancers12071968

**Published:** 2020-07-20

**Authors:** Won-Mook Choi, Danbi Lee, Ju Hyun Shim, Kang Mo Kim, Young-Suk Lim, Han Chu Lee, Changhoon Yoo, Sook Ryun Park, Min-Hee Ryu, Baek-Yeol Ryoo, Jonggi Choi

**Affiliations:** 1 Department of Gastroenterology, Liver Center, Asan Medical Center, University of Ulsan College of Medicine, Seoul 05505, Korea; j.choi@amc.seoul.kr (W.-M.C.); leighdb@hanmail.net (D.L.); s5854@amc.seoul.kr (J.H.S.); kimkm70@amc.seoul.kr (K.M.K.); limys@amc.seoul.kr (Y.-S.L.); hch@amc.seoul.kr (H.C.L.); 2 Department of Oncology, Asan Medical Center, University of Ulsan College of Medicine, Seoul 05505, Korea; yooc@amc.seoul.kr (C.Y.); srpark@amc.seoul.kr (S.R.P.); miniryu@amc.seoul.kr (M.-H.R.); ryooby@amc.seoul.kr (B.-Y.R.)

**Keywords:** liver cancer, immune checkpoint inhibitor, effectiveness, safety

## Abstract

Nivolumab has shown durable response and safety in patients with hepatocellular carcinoma (HCC) in previous trials. However, real-world data of nivolumab in HCC patients, especially those with Child–Pugh class B, are limited. To investigate the effectiveness and safety of nivolumab in a real-world cohort of patients with advanced HCC, we retrospectively evaluated 203 patients with HCC who were treated with nivolumab between July 2017 and February 2019. Of 203 patients, 132 patients were classified as Child–Pugh class A and 71 patients were Child–Pugh class B. Objective response rate was lower in patients with Child–Pugh class B than A (2.8% vs. 15.9%; *p* = 0.010). Child–Pugh class B was an independent negative predictor for objective response. Median overall survival was shorter in Child–Pugh B patients (11.3 vs. 42.9 weeks; adjusted hazard ratio [AHR], 2.10; *p* < 0.001). In Child–Pugh B patients, overall survival of patients with Child–Pugh score of 8 or 9 was worse than patients with Child–Pugh score of 7 (7.4 vs. 15.3 weeks; AHR, 1.93; *p* < 0.020). In conclusion, considering the unsatisfactory response in Child–Pugh B patients, nivolumab may not be used in unselected Child–Pugh B patients. Further studies are needed in this patient population.

## 1. Introduction

Hepatocellular carcinoma (HCC) is the sixth most prevalent cancer, and the second most common cause of cancer deaths in Korea and worldwide, leading to nearly 745,000 deaths globally each year [1,2]. Many patients are newly diagnosed with advanced HCC despite regular surveillance of patients at risk, and disease recurrence or progression after initial treatment, which requires systemic therapy, is common [3,4]. Sorafenib, an oral multikinase inhibitor which improved overall survival (OS) compared to placebo in the SHARP trial [5], has been the only viable treatment for HCC over the last decade, but recent successful phase 2/3 trials of first- or second-line therapies have expanded the treatment landscape for patients with advanced HCC [6,7,8,9]. However, the majority of systemic therapies have been studied in Child–Pugh A populations. Because most trials of systemic therapies for HCC excluded patients with poor liver function (Child–Pugh B or greater hepatic dysfunction). Therefore, limited data are currently available for systemic therapies in patients with advanced liver cirrhosis. In real-world practice, liver function of patients with advanced HCC who require systemic therapy is often poor due to the tumor itself, or it has been deteriorated by previous treatments for HCC. Thus, real-world data regarding the safety and clinical outcomes of systemic therapy in HCC patients with poor liver function are of importance to guide the use of systemic therapy in this population.

Nivolumab, an immune checkpoint inhibitor that blocks programmed cell death protein-1, showed durable responses and prolonged long-term survival in the CheckMate 040 trial [6]. Although patients with Child–Pugh class B disease were included in the CheckMate 040 study, the efficacy and safety of nivolumab have not been established in HCC patients with advanced cirrhosis. Eligibility was restricted to patients with Child–Pugh scores of 7 or 8 and patients with ascites requiring paracentesis were excluded from the study. A few retrospective cohort studies and case series have reported on the safety and effectiveness of immune checkpoint inhibitors for advanced HCC patients with poor liver function [10,11,12]; however, those studies were limited by small numbers of patients. Here we report real-world data on the clinical outcomes and safety of nivolumab using a large retrospective cohort of patients with advanced HCC, including a large number of Child–Pugh B patients.

## 2. Results

### 2.1. Baseline Characteristics of the Study Cohort

A total of 203 patients were included in the study. Information on patient demographics, liver function characteristics, and cancer staging is presented in Table 1. Of the included patients, 132 patients had Child–Pugh class A disease and 71 patients had Child–Pugh class B disease. Most of the baseline characteristics between Child–Pugh A and B patients were significantly different. The Eastern Cooperative Oncology Group (ECOG) performance status was higher in Child–Pugh B patients than in Child–Pugh A patients. Moreover, Child–Pugh B patients had more aggressive tumor characteristics at baseline than Child–Pugh A patients, including higher levels of α-fetoprotein and protein induced by vitamin K absence or antagonist-II, and Child–Pugh B patients had more patients with portal vein invasion (Table 1).

### 2.2. Treatment Outcomes of Patients Receiving Nivolumab

Over a maximum follow-up period of 37.0 months with a median follow-up duration of 5.6 months (interquartile range [IQR], 2.3–11.4), 146 patients died, and 150 patients experienced disease progression after nivolumab treatment. The treatment overview of the study population is summarized in Table 2. The median duration of nivolumab treatment was 1.6 (IQR, 0.9–5.0) and 0.9 (IQR, 0.5–1.9) months with a median of four (ranged 1–57) and three (ranged 1–34) cycles; 17 (12.9%) and eight (11.3%) patients remained on treatment at the time of the last follow-up in Child–Pugh A and B groups, respectively. One hundred and fifteen (87.1%) and 63 (88.7%) patients discontinued treatment in Child–Pugh A and B groups, respectively; the reasons for treatment discontinuation were disease progression in 103 (78.0%) and 46 (64.8%) patients, death in seven (5.3%) and 16 (22.5%) patients, and adverse events (AEs) in five (3.8%) and one (1.4%) patients in Child–Pugh A and B groups, respectively. During treatment, one patient (0.5%) achieved a complete response, while 22 (10.8%) patients achieved a partial response, and 49 (24.1%) patients had stable disease, with an 11.3% objective response rate (ORR) in the total study population.

### 2.3. Treatment Outcomes Stratified by Child–Pugh Class

When stratified by Child–Pugh class, ORR was significantly lower in the Child–Pugh B group than in the Child–Pugh A group (2.8% vs. 15.9%; *p* = 0.010) (Table 3). Two Child–Pugh B patients (2.8%) achieved partial response, with response ongoing for over six months at the time of last follow-up. Disease control rate (DCR) in the total study population was 35.5%. DCR was lower in the Child–Pugh B group than in the Child–Pugh A group (22.5% vs. 42.4%; *p* = 0.008) (Table 3).

**Figure 1:**
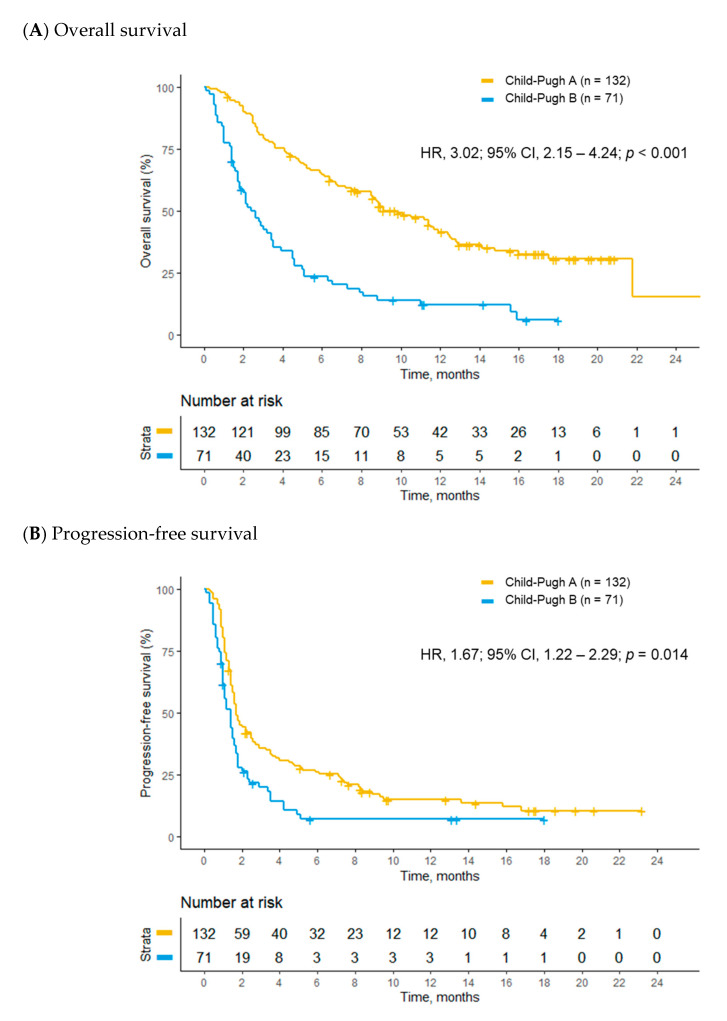
Kaplan–Meier analyses of survival outcomes between Child–Pugh A and B patients. (**A**) overall survival and (**B**) progression-free survival.

OS was longer in the Child–Pugh A group than in the Child–Pugh B group (42.9 vs. 11.3 weeks; hazard ratio [HR], 3.02; 95% confidence interval [CI], 2.15–4.24; *p* < 0.001; Figure 1A); consistent results were also seen in the multivariable analyses (adjusted hazard ratio [AHR], 2.10; 95% CI, 1.38–3.19; *p* < 0.001) (Table 4). In addition to Child–Pugh class, ECOG performance status, albumin-bilirubin grade of 3, and α-fetoprotein were other independent prognostic factors for OS of the study population in the multivariable analysis (Table S1). Median progression-free survival (PFS) was longer in the Child–Pugh A group than in the Child–Pugh B group in the univariate analysis (7.4 vs. 6.0 weeks; HR, 1.67; 95% CI, 1.22–2.29; *p* = 0.014; Figure 1B); however, this difference was not statistically significant after multivariable adjustment (AHR, 1.17; 95% CI, 0.79–1.72; *p* = 0.430) (Table 4). ECOG performance status and liver involvement of HCC were poor prognostic factors for PFS in the multivariable analysis (Table S2). Median time to progression (TTP) was 7.9 weeks (95% CI, 7.1–11.6) for the Child–Pugh A group and 6.9 weeks (95% CI, 6.0–10.1) for the Child–Pugh B group. There was no difference in TTP between the two groups in the univariate (HR, 1.35; 95% CI, 0.95–1.92; *p* = 0.093) and multivariable (AHR, 1.00; 95% CI, 0.66–1.50; *p* = 0.992) analyses (Table 4).

### 2.4. Treatment Outcomes of Child–Pugh B Patients Receiving Nivolumab

**Figure 2:**
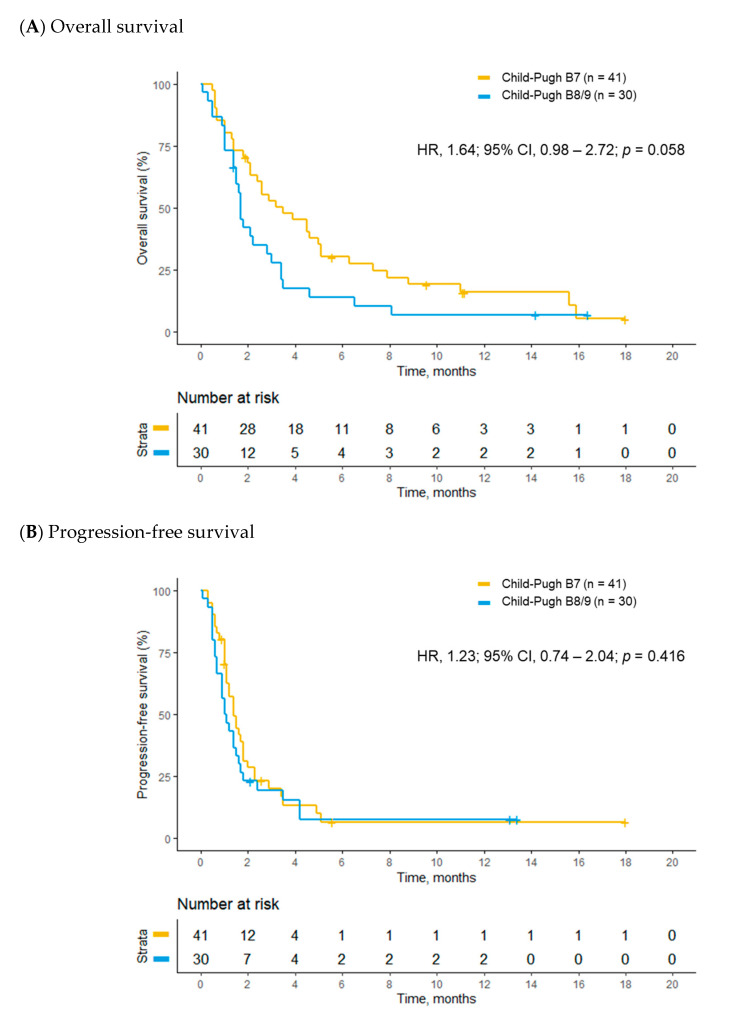
Kaplan–Meier analyses of survival outcomes between Child–Pugh B7 and B8/9 patients. (**A**) overall survival and (**B**) progression-free survival.

Of 71 patients with Child–Pugh class B disease, 41 patients had a Child–Pugh score of 7 and the remaining 30 patients had Child–Pugh scores of 8 or 9. Marginally longer OS was observed in patients with a Child–Pugh score of 7 compared to patients with Child–Pugh scores of 8 or 9 in the univariate analysis (15.3 vs. 7.4 weeks; HR, 1.64; 95% CI, 0.98–2.72; *p* = 0.058; Figure 2A), and this difference became statistically significant after multivariable adjustment (AHR, 1.93; 95% CI, 1.11–3.35; *p* = 0.020) (Table 5). There were no significant differences in PFS (6.3 vs. 4.8 weeks; HR, 1.23; 95% CI, 0.74–2.04; *p* = 0.416; Figure 2B and AHR, 1.53; 95% CI, 0.86–2.58; *p* = 0.153) and TTP (6.9 vs. 6.1 weeks; HR, 1.04; 95% CI, 0.57–1.88; *p* = 0.895 and AHR, 1.30; 95% CI, 0.70–2.40; *p* = 0.408) between the two groups both in the univariate and multivariable analyses (Table 5). ECOG performance status and lung involvement of HCC were independent risk factors for poor OS and PFS in the multivariate analysis (Tables S3 and S4).

### 2.5. Predictive Factors Associated with Treatment Response

Regarding predictive factors associated with treatment response (i.e., complete response and partial response) in patients receiving nivolumab, patients with advanced liver disease (Child–Pugh class B vs. Child–Pugh class A), high levels of tumor markers and liver involvement of HCC were poorly responsive to treatment by univariate analysis. After inclusion of predictive factors with a *p* value < 0.05 from the univariate analysis in the multivariable-adjusted model, Child–Pugh class (B vs. A; adjusted odds ratio [AOR], 0.21; 95% CI, 0.05–0.93; *p* = 0.040) and liver involvement of HCC (AOR, 0.34; 95% CI, 0.13–0.92; *p* = 0.034) remained as significant independent negative predictors for treatment response (Table 6).

Considering the low response rate in Child–Pugh class B patients, predictive factors associated with disease control (i.e., complete response, partial response, and stable disease) were assessed instead of treatment response. Characteristics associated with higher tumor burden including the presence of extrahepatic metastasis, lung involvement of HCC, and ≥ 3 numbers of involved disease sites were significant negative predictors for disease control in the univariate analysis. Among them, lung involvement of HCC (AOR, 0.14; 95% CI, 0.03–0.64; *p* = 0.011) remained a significant independent negative predictor for disease control in the multivariable analysis (Table S5).

### 2.6. Safety of Nivolumab

During the treatment, five (3.8%) patients in the Child–Pugh A group and one (1.4%) patient in the Child–Pugh B group had grade 3 or higher toxicities that were probably attributable to nivolumab, leading to drug discontinuation. In the Child–Pugh A group, two patients developed immune-mediated hepatitis, three patients developed immune-mediated pneumonitis. One patient in the Child–Pugh B group suffered from severe anorexia (Table 7). Eleven (8.3%) and 11 (15.5%) patients in the Child–Pugh A and Child–Pugh B groups, respectively, required dose delay due to AEs (Table 7).

## 3. Discussion

We evaluated the effectiveness and safety of nivolumab in a large real-world cohort of advanced HCC patients including Child–Pugh B patients. ORR and DCR were lower in Child–Pugh B patients than in Child–Pugh A patients and Child–Pugh class B was an independent negative predictor for objective response in our patients. OS was shorter in Child–Pugh B patients. However, TTP and PFS were comparable between Child–Pugh A and B patients by multivariable-adjusted analysis. In the subgroup analysis of Child–Pugh B patients, patients with a Child–Pugh score of 7 survived longer than patients with Child–Pugh scores of 8 or 9; however, there were no differences in PFS and TTP between the two groups. Regarding predictors for nivolumab response in Child–Pugh B patients, lung involvement of HCC, which might represent tumor spread and burden based on the fact that most of the Child–Pugh B patients in our study had liver involvement of HCC, was the only significant negative predictor for disease control. No significant differences were observed in the safety measures of nivolumab between the two groups. Rather, immune-mediated serious AEs due to nivolumab treatment were found to occur less frequently in Child–Pugh B patients than in Child–Pugh A patients.

A lower ORR and DCR in Child–Pugh B patients compared with Child–Pugh A patients observed in our study can be interpreted in two ways. First, Child–Pugh B patients received fewer cycles and had shorter durations of nivolumab treatment than Child–Pugh A patients. Indeed, 22.5% of Child–Pugh B patients discontinued treatment due to death mostly resulting from liver function deterioration, whereas only 5.3% of Child–Pugh A patients ceased the treatment due to death. These facts imply that some patients with poor liver function may not have had enough time to maintain nivolumab treatment because of progressive liver dysfunction. Moreover, these may adversely affect the overall poorer outcomes in Child–Pugh B patients compared with Child–Pugh A patients.

Second, it is well-established that cirrhosis is associated with innate and adaptive immune dysfunction. Moreover, the immune function becomes more impaired as underlying liver cirrhosis progresses [13]. A previous study showed that the cyclooxygenase-derived prostaglandin E2 drives cirrhosis-associated immunosuppression [14]. In addition, patients with decompensated cirrhosis are more vulnerable to endotoxemia or bacteremia, resulting in the up-regulation of prostaglandin E2, and comorbidity with hypoalbuminemia in these patients also provokes increased levels of free prostaglandin E2, causing pathological immune impairment [15]. Cirrhosis alters the number and function of monocytes, NK cells, and T lymphocytes, which play a key role in killing tumor cells [13,16,17]. Thus, proper tumor-killing may not be possible even if T cell reinvigoration is induced by nivolumab, which may explain the poorer ORR in Child–Pugh B patients than in Child–Pugh A patients. A lower incidence of immune-mediated AEs in Child–Pugh B patients also supports this hypothesis.

Recently, results of clinical trials evaluating the efficacy and safety of immune checkpoint inhibitors alone or combination therapies for HCC have been published. In the KEYNOTE-240 trial, although pembrolizumab in a second-line setting after prior sorafenib therapy improved OS (HR, 0.78; 95% CI, 0.61–1.00; one-sided *p* = 0.024) and PFS (HR, 0.72; 95% CI, 0.57–0.90; one-sided *p* = 0.002) compared to placebo, the outcomes did not reach statistical significance per specified criteria [18]. In the CheckMate-459 trial, nivolumab showed an improved OS compared to sorafenib; however, this difference also was not statistically significant [19]. As a combination therapy, atezolimumab with bevacizumab led to better OS (HR, 0.58; 95% CI, 0.42–0.79) than sorafenib in patients with unresectable HCC [20]. However, none of those clinical trials included Child–Pugh class B patients due to competing risk of death from underlying cirrhosis. Moreover, most of the ongoing clinical trials of immune checkpoint inhibitors target Child–Pugh class A disease.

There is insufficient evidence for the use of systemic therapy in Child–Pugh class B patients. The most widely reported systemic therapy in this population is sorafenib. A meta-analysis of thirty studies demonstrated that Child–Pugh B liver function is associated with worse OS compared to Child–Pugh A liver function despite similar response rate, safety, and tolerability [21]. Several previous studies have evaluated the efficacy and safety of nivolumab in Child–Pugh class B patients. In the Child–Pugh B cohort of CheckMate 040 trial, outcomes were much better than was seen in our patients: median OS was 7.6 months, ORR was 10.2%, and the DCR was 55.1% [22]. However, it is important to note that the CheckMate 040 Child–Pugh B cohort excluded patients with Child–Pugh scores of 9 points and patients with ECOG performance status 2 or recent history of paracentesis for ascites or hepatic encephalopathy. In contrast, the current study cohort included patients with more advanced disease with a Child–Pugh score of 9 reported in approximately 21.1% of patients and ECOG performance status 2 reported in approximately 16.9% of patients, thus providing a real-world data of the effectiveness and safety of nivolumab in a wider range of patients. Several retrospective case series or cohort studies of Child–Pugh B patients reported better ORR, from 11.8% to 20%, and longer median OS, from 5.9 to 8.6 months, compared to our Child–Pugh B cohort [10,11,12,23]. However, it is questionable how the ORR in those retrospective studies was better than in the CheckMate 040 Child–Pugh B cohort, notwithstanding the fact that patients with more advanced liver disease were included and the tumor response was evaluated by Response Evaluation Criteria in Solid Tumors (RECIST) 1.1 criteria instead of modified RECIST (mRECIST) in those retrospective studies. Selection bias might also be an issue because the previous retrospective studies had very small patient numbers. Besides, the patients included in our study had more aggressive tumor features with higher proportions of macroscopic vascular invasion and extrahepatic spread compared to the patients included in the previous studies.

Adverse effects of immune checkpoint inhibitors such as nivolumab are different from those of systemic chemotherapy. Clinicians should be aware of immune-mediated AEs such as immune-mediated hepatitis and pneumonitis when using nivolumab. As observed in the clinical trials and previous studies, the AEs of nivolumab in our cohort were manageable and nivolumab appeared to be safe even in Child–Pugh B patients overall. Grade 1 or 2 AEs occurred more frequently in Child–Pugh B patients but were attributed to comorbid liver disease rather than nivolumab treatment. Interestingly, we observed a lower incidence of immune-mediated AEs in Child–Pugh B patients compared to Child–Pugh A patients albeit the incidence of immune-mediated AEs was very low.

This study had several limitations. First, as a retrospective study, it has inherent limitations including bias and confounding. Considering that most clinical trials only include patients with Child–Pugh class A to avoid competing risks of death from liver cirrhosis on the overall outcome, this retrospective cohort study may provide valuable information for evaluating the effectiveness and safety of nivolumab in a real-world setting where the patients tend to be more heterogeneous than patients in clinical trials. Second, as a single-center study, this study may have limited generalizability. Third, most of the HCC cases were caused by hepatitis B virus infection, which may be associated with poorer prognosis [24]. However, there was no evidence that underlying HCC etiology affects the efficacy of nivolumab treatment [25]. Finally, since the data were collected retrospectively from electronic medical records, only adverse events resulting in drug discontinuation or dose delay could be identified in detail. However, considering that nivolumab was well-tolerated, with the exception of rarely occurring severe immune-mediated adverse events seen in previous studies [6,10,11,12,18,21], we believe that the information on adverse events of our study contains clinically meaningful information despite the lack of detailed adverse event information.

## 4. Materials and Methods

### 4.1. Study Population

From July 2017 to February 2019, 221 consecutive patients received nivolumab treatment for unresectable HCC at Asan Medical Center and were retrospectively enrolled in this study. HCC diagnosis was based on multiphase computed tomography and/or magnetic resonance imaging or pathological confirmation in selected cases according to the current international guidelines of HCC [26]. Patients were excluded if they had Child–Pugh class C liver function (*n* = 5), had ECOG performance status > 2 (*n* = 2), had received liver transplantation (*n* = 5), or had been followed-up for less than one cycle of nivolumab (*n* = 6). After excluding 18 patients, 203 patients were included in the final analyses. Nivolumab was administered at 3 mg/kg body weight every 2 weeks intravenously until disease progression, severe adverse events, or death occurred. Dosage delays were permitted according to individual patient tolerability. Patient information, including demographic characteristics, laboratory results, safety assessment and grading, and clinical outcomes were collected from electronic medical records. The response evaluation was carried out every 6-8 weeks during nivolumab treatment, and additional image examinations were allowed when clinically indicated.

This study was approved by the Institutional Review Board of Asan Medical Center (IRB No. 2019-0605) and the informed consent of enrolled patients was waived owing to the retrospective nature of the study.

### 4.2. Outcome Assessment

Clinical tumor response was assessed by the mRECIST criteria [27]. ORR, defined as the proportion of patients with complete or partial response, and DCR, defined as the proportion of patients with complete response, partial response, or stable disease, were evaluated. Other oncological outcomes included TTP, defined as the time from nivolumab treatment to radiological or clinical progression; PFS, defined as the time from nivolumab treatment to progression or death due to any cause; and OS, defined as the time from nivolumab treatment to death due to any cause. Safety assessment and grading were recorded in patients’ electronic medical records when treatment-related adverse events led to dose reduction or discontinuation of nivolumab.

### 4.3. Statistical Analysis

Categorical variables were analyzed as frequency and percentages and were compared using Fisher’s exact test or the chi-square test, as appropriate. Continuous variables were expressed as median and IQR or mean and standard deviation and were compared using unpaired two-tailed *t* tests. Survival outcomes were estimated by the Kaplan–Meier method and compared with the log-rank test. In addition, univariate and multivariable Cox proportional hazard models were used to calculate HRs for survival outcomes and their 95% CIs. To identify the predictive factors associated with treatment response of nivolumab, univariate and multivariable logistic regression models were applied. Variables with *p* values less than 0.05 in the univariate analysis were used in the multivariable analysis.

All statistical analyses were performed using R statistical software, version 3.6.1 (R Foundation Inc; http://cran.r-project.org/). For all analyses, *p* values < 0.05 were considered statistically significant.

## 5. Conclusions

In the present study, the ORR and DCR were lower and the OS was shorter in Child–Pugh class B patients than those in Child–Pugh class A patients. Moreover, Child–Pugh class B was an independent negative predictor for nivolumab response. In particular, Child–Pugh B patients who had high tumor burden with lung involvement or Child–Pugh scores of 8 or 9 may not benefit from nivolumab treatment. Considering the unsatisfactory treatment response and poor prognosis in Child–Pugh B patients, nivolumab may not be beneficial in unselected patients of this patient population. Further investigation in this patient population is needed to confirm our findings.

## Figures and Tables

**Table 1:** Baseline characteristics of the study population.

Characteristics	Child–Pugh A (*n* = 132)	Child–Pugh B (*n* = 71)	*p* Value
Demographics			
Age, mean ± SD, y	56.9 ± 11.2	56.0 ± 9.4	0.576
Male sex, *n* (%)	115 (87.1)	56 (78.9)	0.182
Etiology, HBV/HCV/Other, *n* (%)	111/4/17 (84.1/3.0 /12.9)	57/4/10 (80.3/5.6/14.1)	0.630
ECOG performance status, *n* (%)			0.004
0/1/2	64/57/11 (48.5/43.2/8.3)	18/41/12 (25.4/57.7/16.9)	
Tumor characteristics			
BCLC stage, *n* (%)			0.822
Intermediate/Advanced	6/126 (4.5/95.5)	2/69 (2.8/97.2)	
Portal vein invasion, *n* (%)	46 (34.8)	42 (59.2)	0.001
Extrahepatic metastasis, *n* (%)	120 (90.9)	64 (90.1)	>0.999
Involved disease sites, *n* (%)			
Liver	104 (78.8)	66 (93.0)	0.016
Lung	79 (59.8)	48 (67.6)	0.349
Number of involved disease sites, *n* (%)			0.959
1–2/≥3	80/52 (60.6/69.4)	42/29 (59.2/40.8)	
α-Fetoprotein, median (IQR), ng/mL	311 (10, 3392)	2698 (44, 53727)	0.001
PIVKA-II, median (IQR), mAU/mL	1439 (150, 9129)	6846 (771, 57522)	<0.001
Immunotherapy as systemic, *n* (%)			0.218
First-line	2 (1.5)	1 (1.4)	
Second-line	89 (67.4)	56 (78.9)	
Third-line or more	41 (31.1)	14 (19.7)	
Liver function			
Child–Pugh score, *n* (%)			–
5/6	67/65 (50.8/40.2)	–	
7/8/9	–	41/15/15 (57.7/21.2/21.2)	
Platelet count, *n* (%) ≥150,000/μL <150,000/μL	65 (40.2) 67 (50.8)	26 (36.6) 45 (63.4)	0.115
Ascites, present, *n* (%)	6 (4.5)	48 (67.6)	<0.001
Albumin, median (IQR), g/dL	3.6 (3.2, 3.9)	2.9 (2.6, 3.2)	<0.001
>3.5/2.8–3.5/<2.8, *n* (%)	81/51/0 (61.4/38.6 /0.0)	8/33/30 (11.3/46.5/42.3)	
Total bilirubin, median (IQR), mg/dL	0.7 (0.5, 0.9)	1.3 (0.9, 2.0)	<0.001
<2/2–3 />3, *n* (%)	130/2/0 (98.5/1.5/0.0)	52/12/7 (73.2/16.9/9.9)	
ALBI grade, mean ± SD	−2.33 ± 0.37	−1.54 ± 0.37	<0.001
1/2/3, *n* (%)	35/97/0 (26.5/73.5/0.0)	0/49/22 (0.0/69.0/31.0)	

Abbreviations: ALBI, albumin-bilirubin; BCLC, Barcelona Clinic Liver Cancer; ECOG, Eastern Cooperative Oncology Group; HBV, hepatitis B virus; HCV, hepatitis C virus; IQR, interquartile range; PIVKA, protein induced by vitamin K absence or antagonist-II; SD, standard deviation.

**Table 2:** Treatment summary of the study population.

Disposition Characteristics	Child–Pugh A (*n* = 132)	Child–Pugh B (*n* = 71)
Treatment duration, months (IQR)	1.6 (0.9, 5.0)	0.9 (0.5, 1.9)
Treatment duration, cycles, median (range) mean ± SD	4 (1–57) 8.5 ± 9.9	3 (1–34) 4.3 ± 5.3
Continuing treatment, *n* (%)	17 (12.9)	8 (11.3)
Discontinued treatment, *n* (%)	115 (87.1)	63 (88.7)
Disease progression, *n* (%)	103 (78.0)	46 (64.8)
Death, *n* (%)	7 (5.3)	16 (22.5)
Adverse events, *n* (%)	5 (3.8)	1 (1.4)

Abbreviations: IQR, interquartile range; SD, standard deviation.

**Table 3:** Tumor responses in the study population according to modified Response Evaluation Criteria in Solid Tumors (mRECIST) criteria.

Entire Cohort			
Tumor Responses	Child–Pugh A (*n* = 132)	Child–Pugh B (*n* = 71)	*p* Value ^a^
Best overall response, *n* (%)			
Complete response	1 (0.8)	0 (0.0)	
Partial response	20 (15.2)	2 (2.8)	
Stable disease	35 (26.5)	14 (19.7)	
Progressive disease	69 (52.3)	40 (56.3)	
Not evaluable ^b^	7 (5.3)	15 (21.1)	
Objective response ^c^, *n* (%)	21 (15.9)	2 (2.8)	0.010
Disease control rate ^d^, *n* (%)	56 (42.4)	16 (22.5)	0.008

^a^ By χ^2^ test or Fisher exact test, as appropriate, for radiologic response. ^b^ Due to death without radiologic disease progression or early drug discontinuation due to a severe adverse drug reaction. ^c^ Objective response rate, defined as the proportion of patients who had complete response or partial response. ^d^ Disease control rate, defined as the proportion of patients who had complete response, partial response, or stable disease.

**Table 4:** Survival outcomes of the study population.

Entire Cohort						
Outcome	Median Time, Week (95% CI)		Univariate Analysis		Multivariable Analysis	
	Child–Pugh A (*n* = 132)	Child–Pugh B (*n* = 71)	HR (95% CI)^a^	*p* Value	AHR (95% CI) ^a^	*p* Value
Overall survival	42.9 (34.1–54.3)	11.3 (7.7–15.4)	3.02 (2.15–4.24)	<0.001	2.10 (1.38–3.19)	<0.001
Progression-free survival	7.4 (7.0–11.0)	6.0 (4.7–7.6)	1.67 (1.22–2.29)	0.014	1.17 (0.79–1.72)	0.430
Time to progression	7.9 (7.1–11.6)	6.9 (6.0–10.1)	1.35 (0.95–1.92)	0.093	1.04 (0.72–1.51)	0.834

^a^ Cox proportional hazard regression model for the Child–Pugh B group with the Child–Pugh A group as a reference. Abbreviations: AHR, adjusted hazard ratio; CI, confidence interval; HR, hazard ratio.

**Table 5:** Survival outcomes of Child–Pugh B patients.

Entire Cohort						
Outcome	Median Time, Week (95% CI)		Univariate Analysis		Multivariable Analysis	
	Child–Pugh B7 (*n* = 41)	Child–Pugh B8/9 (*n* = 30)	HR (95% CI) ^a^	*p* Value	AHR (95% CI) ^a^	*p* Value
Overall survival	15.3 (9.3–22.3)	7.4 (6.4–14.9)	1.64 (0.98–2.72)	0.058	1.93 (1.11–3.35)	0.020
Progression-free survival	6.3 (5.0–8.0)	4.8(3.7–7.6)	1.23 (0.74–2.04)	0.416	1.53 (0.86–2.58)	0.153
Time to progression	6.9 (6.0–12.6)	6.1 (4.6–NA)	1.04 (0.57–1.88)	0.895	1.30 (0.70–2.40)	0.408

^a^ Cox proportional hazard regression model for the Child–Pugh B8/9 group with the Child–Pugh B7 group as a reference. Abbreviations: AHR, adjusted hazard ratio; CI, confidence interval; HR, hazard ratio; NA, not applicable.

**Table 6:** Predictive factors for treatment response.

Characteristics	Univariate Analysis		Multivariable Analysis	
	OR (95% CI)	*p* Value	AOR (95% CI)	*p* Value
Child–Pugh class A B	1 (reference) 0.15 (0.03–0.67)	0.013 *	1 (reference) 0.21 (0.05–0.93)	0.040
Age	1.02 (0.98–1.07)	0.305	-	-
Sex Female Male	1 (reference) 2.10 (0.47–9.43)	0.333	-	-
Ascites, present	0.23 (0.05–1.04)	0.056	-	-
α-Fetoprotein, ng/mL <400 ≥400	1 (reference) 0.66 (0.27–1.58)	0.349	-	-
PIVKA-II, mAU/mL <2000 ≥2000	1 (reference) 0.37 (0.14–0.93)	0.035	1 (reference) 0.55 (0.21–1.47)	0.234
Albumin (per 1 g/dL increase)	1.96 (0.83–4.62)	0.125	-	-
Total bilirubin (per 1 mg/dL increase)	0.82 (0.48–1.40)	0.469	-	-
ALBI grade 1 2 3	1 (reference) 0.79 (0.27–2.31) 0.29 (0.03–2.63)	0.668 0.268	-	-
Etiology HBV Non-HBV etiology	1 (reference) 2.38 (0.90–6.30)	0.082	-	-
Portal vein invasion, present	0.53 (0.21–1.36)	0.190	-	-
Extrahepatic metastasis, present	2.44 (0.31–19.22)	0.396	-	-
Involved disease sites, present				
Liver	0.24 (0.09–0.61)	0.003	0.34 (0.13–0.92)	0.034
Lung	1.14 (0.46–2.83)	0.780	-	-
Number of involved disease sites per patient 1–2 ≥3	1 (reference) 0.38 (0.14–1.07)	0.067	-	-

Abbreviations: AOR; adjusted odds ratio; ALBI, albumin-bilirubin; CI, confidence interval; HBV, hepatitis B virus; OR, odds ratio; PIVKA, protein induced by vitamin K absence or antagonist-II. * *p* < 0.05.

**Table 7:** Adverse events requiring discontinuation or dose delay.

Adverse Events	Child–Pugh A (*n* = 132)		Child–Pugh B (*n* = 71)	
	Any Grade	Grade ≥ 3	Any Grade	Grade ≥ 3
Hepatitis	3 (2.3)	2 (1.5)	3 (4.2)	
Pneumonitis	3 (2.3)	3 (2.3)		
Anorexia	3 (2.3)		6 (8.5)	1 (1.4)
Nausea	1 (0.8)		1 (1.4)	
Pain	1 (0.8)		3 (4.2)	
Anemia	3 (2.3)		3 (4.2)	
Fatigue	1 (0.8)		5 (7.0)	
Rash	2 (1.5)		1 (1.4)	
Insomnia			1 (1.4)	

## Supplementary Materials

The following are available online at https://www.mdpi.com/2072-6694/12/7/1968/s1, Table S1: Univariate and multivariable analyses for overall survival of the study population, Table S2: Univariate and multivariable analyses for progression-free survival of the study population, Table S3: Univariate and multivariable analyses for overall survival of Child-Pugh B patients, Table S4: Univariate and multivariable analyses for progression-free survival of Child-Pugh B patients, Table S5: Predictive factors for disease control in Child-Pugh B patients.

## Abbreviations

AE	adverse event
AHR	adjusted hazard ratio
AOR	adjusted odds ratio
CI	confidence interval
DCR	disease control rate
ECOG	Eastern Cooperative Oncology Group
HCC	hepatocellular carcinoma
HR	hazard ratio
IQR	interquartile range
mRECIST	modified Response Evaluation Criteria in Solid Tumors
OS	overall survival
ORR	objective response rate
PFS	progression-free survival
RECIST	Response Evaluation Criteria in Solid Tumors
TTP	time to progression

## References

[1] Bray F., Ferlay J., Soerjomataram I., Siegel R.L., Torre L.A., Jemal A. (2018). Global cancer statistics 2018: GLOBOCAN estimates of incidence and mortality worldwide for 36 cancers in 185 countries. CA Cancer J. Clin..

[2] Kim B.H., Park J.W. (2018). Epidemiology of liver cancer in South Korea. Clin. Mol. Hepatol..

[3] Vogel A., Cervantes A., Chau I., Daniele B., Llovet J.M., Meyer T., Nault J.C., Neumann U., Ricke J., Sangro B. (2018). Hepatocellular carcinoma: ESMO Clinical Practice Guidelines for diagnosis, treatment and follow-up. Ann. Oncol..

[4] Pinter M., Peck-Radosavljevic M. (2018). Review article: Systemic treatment of hepatocellular carcinoma. Aliment. Pharmacol. Ther..

[5] Llovet J.M., Ricci S., Mazzaferro V., Hilgard P., Gane E., Blanc J.F., de Oliveira A.C., Santoro A., Raoul J.L., Forner A. (2008). Sorafenib in advanced hepatocellular carcinoma. N. Engl. J. Med..

[6] El-Khoueiry A.B., Sangro B., Yau T., Crocenzi T.S., Kudo M., Hsu C., Kim T.Y., Choo S.P., Trojan J., Welling T.H.R. (2017). Nivolumab in patients with advanced hepatocellular carcinoma (CheckMate 040): An open-label, non-comparative, phase 1/2 dose escalation and expansion trial. Lancet.

[7] Zhu A.X., Finn R.S., Edeline J., Cattan S., Ogasawara S., Palmer D., Verslype C., Zagonel V., Fartoux L., Vogel A. (2018). Pembrolizumab in patients with advanced hepatocellular carcinoma previously treated with sorafenib (KEYNOTE-224): A non-randomised, open-label phase 2 trial. Lancet Oncol..

[8] Kudo M., Finn R.S., Qin S., Han K.H., Ikeda K., Piscaglia F., Baron A., Park J.W., Han G., Jassem J. (2018). Lenvatinib versus sorafenib in first-line treatment of patients with unresectable hepatocellular carcinoma: A randomised phase 3 non-inferiority trial. Lancet.

[9] Bruix J., Qin S., Merle P., Granito A., Huang Y.H., Bodoky G., Pracht M., Yokosuka O., Rosmorduc O., Breder V. (2017). Regorafenib for patients with hepatocellular carcinoma who progressed on sorafenib treatment (RESORCE): A randomised, double-blind, placebo-controlled, phase 3 trial. Lancet.

[10] Kambhampati S., Bauer K.E., Bracci P.M., Keenan B.P., Behr S.C., Gordan J.D., Kelley R.K. (2019). Nivolumab in patients with advanced hepatocellular carcinoma and Child-Pugh class B cirrhosis: Safety and clinical outcomes in a retrospective case series. Cancer.

[11] Scheiner B., Kirstein M.M., Hucke F., Finkelmeier F., Schulze K., von Felden J., Koch S., Schwabl P., Hinrichs J.B., Waneck F. (2019). Programmed cell death protein-1 (PD-1)-targeted immunotherapy in advanced hepatocellular carcinoma: Efficacy and safety data from an international multicentre real-world cohort. Aliment. Pharmacol. Ther..

[12] Finkelmeier F., Czauderna C., Perkhofer L., Ettrich T.J., Trojan J., Weinmann A., Marquardt J.U., Vermehren J., Waidmann O. (2019). Feasibility and safety of nivolumab in advanced hepatocellular carcinoma: Real-life experience from three German centers. J. Cancer Res. Clin. Oncol..

[13] Albillos A., Lario M., Álvarez-Mon M. (2014). Cirrhosis-associated immune dysfunction: Distinctive features and clinical relevance. J. Hepatol..

[14] O’Brien A.J., Fullerton J.N., Massey K.A., Auld G., Sewell G., James S., Newson J., Karra E., Winstanley A., Alazawi W. (2014). Immunosuppression in acutely decompensated cirrhosis is mediated by prostaglandin E2. Nat. Med..

[15] Choe W.H., Baik S.K. (2015). Prostaglandin E2 -mediated immunosuppression and the role of albumin as its modulator. Hepatology.

[16] Albillos A., Hera Ad Ade L., Reyes E., Monserrat J., Muñoz L., Nieto M., Prieto A., Sanz E., Alvarez-Mon M. (2004). Tumour necrosis factor-alpha expression by activated monocytes and altered T-cell homeostasis in ascitic alcoholic cirrhosis: Amelioration with norfloxacin. J. Hepatol..

[17] Tian Z., Chen Y., Gao B. (2013). Natural killer cells in liver disease. Hepatology.

[18] Finn R.S., Ryoo B.Y., Merle P., Kudo M., Bouattour M., Lim H.Y., Breder V., Edeline J., Chao Y., Ogasawara S. (2020). Pembrolizumab As Second-Line Therapy in Patients with Advanced Hepatocellular Carcinoma in KEYNOTE-240: A Randomized, Double-Blind, Phase III Trial. J. Clin. Oncol..

[19] Yau T., Park J., Finn R., Cheng A.-L., Mathurin P., Edeline J., Kudo M., Han K.-H., Harding J., Merle P. CheckMate 459: A randomized, multi-center phase III study of nivolumab (NIVO) vs sorafenib (SOR) as first-line (1L) treatment in patients (pts) with advanced hepatocellular carcinoma (aHCC). Proceedings of the European Society for Medical Oncology (ESMO) Congress.

[20] Finn R.S., Qin S., Ikeda M., Galle P.R., Ducreux M., Kim T.Y., Kudo M., Breder V., Merle P., Kaseb A.O. (2020). Atezolizumab plus Bevacizumab in Unresectable hepatocellular carcinoma. N. Engl. J. Med..

[21] McNamara M.G., Slagter A.E., Nuttall C., Frizziero M., Pihlak R., Lamarca A., Tariq N., Valle J.W., Hubner R.A., Knox J.J. (2018). Sorafenib as first-line therapy in patients with advanced Child-Pugh B hepatocellular carcinoma-a meta-analysis. Eur. J. Cancer.

[22] Kudo M., Matilla A., Santoro A., Melero I., Gracian A.C., Acosta-Rivera M., Choo S.P., El-Khoueiry A.B., Kuromatsu R., El-Rayes B.F. Checkmate-040: Nivolumab (NIVO) in patients (pts) with advanced hepatocellular carcinoma (aHCC) and Child-Pugh B (CPB) status. Proceedings of the Liver Metting 2018 by Ameircan Association for the Study of Liver Diseases (AASLD).

[23] Lee P.C., Chao Y., Chen M.H., Lan K.H., Lee C.J., Lee I.C., Chen S.C., Hou M.C., Huang Y.H. (2020). Predictors of response and survival in immune checkpoint inhibitor-treated Unresectable hepatocellular carcinoma. Cancers.

[24] Zucman-Rossi J., Villanueva A., Nault J.C., Llovet J.M. (2015). Genetic landscape and biomarkers of hepatocellular carcinoma. Gastroenterology.

[25] Yau T., Hsu C., Kim T.Y., Choo S.P., Kang Y.K., Hou M.M., Numata K., Yeo W., Chopra A., Ikeda M. (2019). Nivolumab in advanced hepatocellular carcinoma: Sorafenib-experienced Asian cohort analysis. J. Hepatol..

[26] Marrero J.A., Kulik L.M., Sirlin C.B., Zhu A.X., Finn R.S., Abecassis M.M., Roberts L.R., Heimbach J.K. (2018). Diagnosis, staging, and management of hepatocellular carcinoma: 2018 practice guidance by the american association for the study of liver diseases. Hepatology.

[27] Lencioni R., Llovet J.M. (2010). Modified RECIST (mRECIST) assessment for hepatocellular carcinoma. Semin. Liver Dis..

