# Supplementary material for: Effectiveness and Safety of Nivolumab in Child–Pugh B Patients with Hepatocellular Carcinoma: A Real-World Cohort Study
Source: Cancers (Basel). 2020 Jul 20;12(7):1968. doi: 10.3390/cancers12071968 (PMC7409289; doi:10.3390/cancers12071968)
Supplement: Supplementary file 1 [file cancers-12-01968-s001.pdf]

cancers

MDPI

Article

Effectiveness and Safety of Nivolumab in Child–Pugh B Patients with Hepatocellular Carcinoma: A Real-World Cohort Study

Won-Mook Choi, Danbi Lee, Ju Hyun Shim, Kang Mo Kim, Young-Suk Lim, Han Chu Lee, Changhoon Yoo, Sook Ryun Park, Min-Hee Ryu, Baek-Yeol Ryoo and Jonggi Choi

Table S1. Univariate and multivariable analyses for overall survival of the study population.

Characteristics	Univariate Analysis		Multivariable Analysis	
	HR (95% CI)	*p* Value	AHR (95% CI)	*p* Value
Child-Pugh class				
A	1 (reference)	0.013	1 (reference)	< 0.001
B	3.02 (2.15–4.24)		2.10 (1.38–3.19)	
Age (per one-year increase)	0.99 (0.98–1.00)	0.191	-	-
Sex				
Female	1 (reference)	0.378	-	-
Male	0.82 (0.53–1.27)			
ECOG				
0	1 (reference)		1 (reference)	
1	2.49 (1.70–3.62)	< 0.001	2.24 (1.50–3.33)	< 0.001
2	7.86 (4.59–13.45)	< 0.001	5.43 (3.10–9.52)	< 0.001
ALBI grade	1 (reference)		1 (reference)	
2	2.23 (1.34–3.72)	0.002	1.49 (0.87–2.56)	0.144
3	4.87 (2.48–9.53)	<0.001	2.51 (1.13–5.57)	0.023
Etiology				
HBV	1 (reference)	0.186	-	-
Non-HBV etiology	0.74 (0.47–1.16)			
BCLC stage				
B	1 (reference)	0.417	-	-
C	1.45 (0.59–3.54)			
α-Fetoprotein, ng/mL				
< 400	1 (reference)	<0.001	1 (reference)	0.008
≥ 400	1.92 (1.37–2.69)		1.62 (1.13–2.31)	
PIVKA-II, mAU/mL				
< 2000	1 (reference)	0.010	1 (reference)	0.943
≥ 2000	1.54 (1.11–2.14)		1.01 (0.71–1.45)	
Portal vein invasion, present	1.69 (1.22–2.35)	0.002	1.17 (0.80–1.72)	0.412
Extrahepatic metastasis, present	0.93 (0.53–1.66)	0.813	-	-
Involved disease sites, present				
Liver	2.51 (1.49–4.22)	<0.001	1.39 (0.76–2.53)	0.283
Lung	1.28 (0.91–1.80)	0.162	-	-
Number of involved disease sites per patient				
1–2	1 (reference)	<0.001	1 (reference)	0.107
≥ 3	1.74 (1.26–2.42)		1.35 (0.94–1.95)	

Abbreviations: AHR; adjusted hazard ratio; ALBI, albumin-bilirubin; BCLC, Barcelona Clinic Liver Cancer; CI, confidence interval; ECOG, Eastern Cooperative Oncology Group; HBV, hepatitis B virus; HR, hazard ratio; PIVKA, protein induced by vitamin K absence or antagonist-II.

Table S2. Univariate and multivariable analyses for progression-free survival of the study population.

**Characteristics**	**Univariate Analysis**		**Multivariable Analysis**	
	**HR (95% CI)**	***p* Value**	**AHR (95% CI)**	***p* Value**
Child-Pugh class				
A	1 (reference)	0.001	1 (reference)	0.430
B	1.67 (1.22–2.29)		1.17 (0.79–1.72)	
Age (per one-year increase)	0.98 (0.97–1.00)	0.017	0.98 (0.97–1.00)	0.048
Sex				
Female	1 (reference)	0.959	-	-
Male	1.01 (0.68–1.50)			
ECOG				
0	1 (reference)		1 (reference)	
1	2.17 (1.55–3.04)	< 0.001	2.07 (1.44–2.98)	< 0.001
2	4.20 (2.52–7.00)	< 0.001	3.22 (1.89–5.49)	< 0.001
ALBI grade	1 (reference)		1 (reference)	
2	1.51 (0.98–2.32)	0.059	1.15 (0.72–1.82)	0.556
3	2.12 (1.16–3.85)	0.014	1.40 (0.67–2.91)	0.370
Etiology				
HBV	1 (reference)	0.143	-	-
Non-HBV etiology	0.73 (0.48–1.11)			
BCLC stage				
B	1 (reference)	0.524	-	-
C	1.30 (0.58–2.95)			
α-Fetoprotein, ng/mL				
< 400	1 (reference)	0.015	1 (reference)	0.751
≥ 400	1.45 (1.08–1.96)		1.05 (0.77–1.45)	
PIVKA-II, mAU/mL				
< 2000	1 (reference)	< 0.001	1 (reference)	0.141
≥ 2000	1.71 (1.27–2.32)		1.28 (0.92–1.77)	
Portal vein invasion, present	1.48 (1.10–2.00)	0.010	0.95 (0.67–1.35)	0.782
Extrahepatic metastasis, present	0.84 (0.50–1.42)	0.520	-	-
Involved disease sites, present				
Liver	2.58 (1.64–4.06)	< 0.001	1.77 (1.04–3.02)	0.036
Lung	1.36 (1.00–1.86)	0.051	-	-
Number of involved disease sites per patient				
1–2	1 (reference)	<0.001	1 (reference)	0.139
≥ 3	1.74 (1.29–2.36)		1.28 (0.92–1.79)	

Abbreviations: AHR; adjusted hazard ratio; ALBI, albumin-bilirubin; BCLC, Barcelona Clinic Liver Cancer; CI, confidence interval; ECOG, Eastern Cooperative Oncology Group; HBV, hepatitis B virus; HR, hazard ratio; PIVKA, protein induced by vitamin K absence or antagonist-II.

Table S3. Univariate and multivariable analyses for overall survival of Child-Pugh B patients.

**Characteristics**	**Univariate Analysis**		**Multivariable Analysis**	
	**HR (95% CI)**	***p* Value**	**AHR (95% CI)**	***p* Value**
Child-Pugh score				
7	1 (reference)	0.058	1 (reference)	0.020
8–9	1.64 (0.98–2.72)		1.93 (1.11–3.35)	
Age (per one-year increase)	0.98 (0.95–1.00)	0.068	-	-
Sex				
Female	1 (reference)	0.659	-	-
Male	1.15 (0.62–2.13)			
ECOG				

0	1 (reference)		1 (reference)	
1	2.18 (1.17–4.07)	0.015	2.10 (1.10–4.01)	0.025
2	3.52 (1.61–7.71)	0.002	3.96 (1.73–9.03)	0.001
ALBI grade				
2	1 (reference)	0.690	-	-
3	1.12 (0.64–1.94)			
Etiology				
HBV	1 (reference)	0.120	-	-
Non-HBV etiology	0.58 (0.29–1.15)			
BCLC stage				
B	1 (reference)	0.875	-	-
C	1.12 (0.27–4.62)			
α-Fetoprotein, ng/mL				
<400	1 (reference)	0.019	1 (reference)	0.307
≥400	1.95 (1.12–3.41)		1.38 (0.74–2.57)	
PIVKA-II, mAU/mL				
<2000	1 (reference)	0.845	-	-
≥2000	1.05 (0.62–1.79)			
Portal vein invasion, present	1.86 (1.09–3.19)	0.024	1.34 (0.74–2.44)	0.331
Extrahepatic metastasis, present	1.28 (0.55–2.99)	0.566	-	-
Involved disease sites, present				
Liver	1.72 (0.62–4.78)	0.297	-	-
Lung	1.77 (1.01–3.09)	0.045	2.20 (1.17–4.13)	0.014
Number of involved disease sites per patient				
1–2	1 (reference)	0.070	-	-
≥3	1.60 (0.96–2.66)			

Abbreviations: AHR; adjusted hazard ratio; ALBI, albumin-bilirubin; BCLC, Barcelona Clinic Liver Cancer; CI, confidence interval; ECOG, Eastern Cooperative Oncology Group; HBV, hepatitis B virus; HR, hazard ratio; PIVKA, protein induced by vitamin K absence or antagonist-II.

Table S4. Univariate and multivariable analyses for progression-free survival of Child-Pugh B patients.

**Characteristics**	**Univariate Analysis**		**Multivariable Analysis**	
	**HR (95% CI)**	***p* Value**	**AHR (95% CI)**	***p* Value**
Child-Pugh score				
7	1 (reference)	0.416	1 (reference)	0.153
8–9	1.23 (0.74–2.04)		1.53 (0.86–2.58)	
Age (per one-year increase)	0.98 (0.95–1.00)	0.102	-	-
Gender				
Female	1 (reference)	0.409	-	-
Male	1.28 (0.71–2.30)			
ECOG				
0	1 (reference)		1 (reference)	
1	1.60 (0.86–2.96)	0.137	1.40 (0.75–2.61)	0.288
2	2.36 (1.06–5.24)	0.036	2.31 (1.03–5.18)	0.042
ALBI grade				
2	1 (reference)	0.789	-	-
3	1.08 (0.63–1.85)			
Etiology				
HBV	1 (reference)	0.279	-	-
Non-HBV etiology	0.70 (0.36–1.34)			
BCLC stage				
B	1 (reference)	0.518	-	-
C	1.92 (0.27–13.92)			
α-Fetoprotein, ng/mL		0.150	-	-

<400	1 (reference)			
≥400	1.47 (0.87–2.48)			
PIVKA-II, mAU/mL				
<2000	1 (reference)	0.326	-	-
≥2000	1.31 (0.77–2.23)			
Portal vein invasion, present	1.92 (1.14–3.25)	0.015	1.44 (0.82–2.51)	0.202
Extrahepatic metastasis, present	1.39 (0.55–3.49)	0.482	-	-
Involved disease sites, present				
Liver	1.85 (0.67–5.13)	0.235	-	-
Lung	2.33 (1.31–4.16)	0.004	2.38 (1.27–4.46)	0.007
Number of involved disease sites per patient				
1–2	1 (reference)	0.100	-	-
≥3	1.52 (0.92–2.52)			

Abbreviations: AHR; adjusted hazard ratio; ALBI, albumin-bilirubin; BCLC, Barcelona Clinic Liver Cancer; CI, confidence interval; ECOG, Eastern Cooperative Oncology Group; HBV, hepatitis B virus; HR, hazard ratio; PIVKA, protein induced by vitamin K absence or antagonist-II.

Table S5. Predictive factors for disease control in Child-Pugh B patients.

Characteristics	Univariate Analysis		Multivariable Analysis	
	OR (95% CI)	*p* Value	AOR (95% CI)	*p* Value
Child-Pugh score				
7	1 (reference)	0.662	-	-
8–9	0.77 (0.25–2.43)			
Age (per one-year increase)	1.05 (0.99–1.12)	0.133	-	-
Gender				
Female	1 (reference)	0.667	-	-
Male	0.75 (0.20–2.78)			
Ascites, present	0.37 (0.12–1.18)	0.093	-	-
α-Fetoprotein, ng/mL				
<400	1 (reference)	0.593	-	-
≥ 400	0.73 (0.24–2.27)			
PIVKA-II, mAU/mL				
<2000	1 (reference)	0.806	-	-
≥2000	1.16 (0.35–3.83)			
Albumin (per 1 g/dL increase)	0.31 (0.08–1.27)	0.104	-	-
Total bilirubin (per 1 mg/dL increase)	0.65 (0.34–1.25)	0.195	-	-
ALBI grade				
2	1 (reference)	0.979	-	-
3	1.02 (0.31–3.38)			
Etiology		0.195		
HBV	1 (reference)		-	-
Non-HBV etiology	2.32 (0.65–8.32)			
Portal vein invasion, present	0.32 (0.10–1.00)	0.051	-	-
Extrahepatic metastasis, present	0.17 (0.03–0.88)	0.034	0.52 (0.06–4.58)	0.557
Involved disease sites, present				
Liver	1.18 (0.12–11.34)	0.888	-	-
Lung	0.13 (0.04–0.44)	0.001	0.14 (0.03–0.64)	0.011
Number of involved disease sites per patient				
1–2	1 (reference)	0.036	1 (reference)	0.856
≥3	0.41 (0.18–0.94)		1.12 (0.33–3.80)	

Abbreviations: AOR; adjusted odds ratio; ALBI, albumin-bilirubin; CI, confidence interval; HBV, hepatitis B virus; OR, odds ratio; PIVKA, protein induced by vitamin K absence or antagonist-II.
